# Apelin-13 alleviated cardiac fibrosis via inhibiting the PI3K/Akt pathway to attenuate oxidative stress in rats with myocardial infarction-induced heart failure

**DOI:** 10.1042/BSR20200040

**Published:** 2020-04-03

**Authors:** Shan Zhong, Hongli Guo, Hui Wang, Dan Xing, Tingting Lu, Jing Yang, Chen Wang

**Affiliations:** 1 Department of Anesthesiology, Children’s Hospital of Nanjing Medical University, Nanjing, China; 2 Department of Anesthesiology, The Affiliated Suzhou Science and Technology Town Hospital of Nanjing Medical University, Suzhou, China; 3 Department of Anesthesiology, Suzhou Hospital (West District) Affiliated to Nanjing Medical University, Suzhou, China

**Keywords:** Apelin-13, fibrosis, heart failure, phosphatidylinositol 3-kinase, protein kinase B, oxidative stress

## Abstract

The present study aimed to determine whether apelin-13 could attenuate cardiac fibrosis via inhibiting the phosphatidylinositol 3-kinase/protein kinase B (PI3K/Akt) pathway to inhibit reactive oxygen species in heart failure (HF) rats. HF models were established by inducing ischemia myocardial infarction (MI) through ligation of the left anterior descending artery in Sprague–Dawley (SD) rats. MI-induced changes in hemodynamics and cardiac function were reversed by apelin-13 administration. The increases in the levels of collagen I, collagen III, α-smooth muscle actin (SMA), and transforming growth factor-β (TGF-β) in the heart of MI rats and cardiac fibroblasts (CFs) treated with angiotensin (Ang) II were inhibited by apelin-13. The levels of PI3K and p-Akt increased in Ang II-treated CFs, and these increases were blocked by apelin-13. The PI3K overexpression reversed the effects of apelin-13 on Ang II-induced increases in collagen I, collagen III, α-SMA, and TGF-β, NADPH oxidase activity and superoxide anions in CFs. Apelin-13 reduced the increases in the levels of NADPH oxidase activity and superoxide anions in the heart of MI rats and CFs with Ang II treatment. The results demonstrated that apelin-13 improved cardiac dysfunction, impaired cardiac hemodynamics, and attenuated fibrosis of CFs induced by Ang II via inhibiting the PI3K/Akt signaling pathway to inhibit oxidative stress.

## Introduction

Chronic heart failure (CHF) is commonly caused by myocardial infarction (MI) [1]. Heart failure (HF) is preceded by ventricular remodelings such as changes in left ventricular (LV) mass and myocardial size after alterations in pressure-overload conditions [2]. Rats with coronary artery ligation-induced HF showed significantly impaired cardiac hemodynamics and cardiac dysfunction [3].

Cardiac fibrosis is a major driver of disease progression in CHF [4], and excessive fibrosis causes large infarct scars, resulting in cardiac dilatation and cardiac dysfunction [5,6]. Cardiomyocytes produce extracellular matrix proteins and therefore contribute to fibrosis. However, resident cardiac fibroblasts (CFs) are currently considered as the main source of fibrosis in the myocardium in response to ischemic injury [7]. CFs played a critical role in postinfarction remodeling, which can ultimately lead to pathological fibrosis and HF [8]. Cardiac fibrosis is a hallmark of HF for which no effective pharmacological therapy is available.

Apelin is a hormone peptide widely found in cardiovascular [9], adipose [10], cerebral [11], and pulmonary tissues [12]. Diverse active apelin peptides exist under the form of 36, 17, or 13 amino acids which originated from a preproproteins consistingof 77 amino acid residues. Apelin-13 has the highest activity of these three active peptides, followed by apelin-17 then apelin-36 [13]. Apelin, together with its receptor APJ, is involved in cardiovascular diseases, diabetes, obesity, and cancer [10,14]. The expression levels of apelin were increased in many pathological states or disease processes such as cardiovascular and metabolic disorders [15]. Increased nitric oxide production through the apelin/APJ/protein kinase B (Akt)/endothelial nitric oxide synthase (eNOS) pathway may, at least in part, contribute to the alleviating effect of losartan in unilateral ureteral obstruction-induced renal fibrosis in mice [16]. Apelin can be an important mediator of fibrogenesis in human liver diseases [17]. However, whether apelin is involved in regulating cardiac fibrosis in MI-induced HF is not well known.

The expression level of phosphatidylinositol 3-kinase (PI3K) and the phosphorylation level of Akt increased in infarcted myocardial tissues of mice [18]. The inhibition of PI3K/Akt signaling activity on treatment with LY294002 markedly reversed the protective effect of erythropoietin on the abdominal aortic constriction-induced myocardial fibrosis [19]. Apelin-13 promotes H9C2 rat cardiomyocyte hypertrophy via the PI3K/Akt signaling pathway and the autophagy induced by PI3K [20]. Angiotensin (Ang) II-stimulated collagen production is mediated through reactive oxygen species (ROS) generation in adult rat CFs. Ang II activates ROS-sensitive kinases that are critical in mediating fibrotic remodeling of the heart [21]. The present study was performed to determine whether apelin-13 improved cardiac function, hemodynamics, and fibrosis in rats with HF and whether apelin-13 attenuated cardiac fibrosis via inhibiting the PI3K/Akt signaling pathway to attenuate oxidative stress.

## Materials and methods

### Animals

The experiments were carried out using male Sprague–Dawley (SD) rats (weighing 160–200 g; Vital River Biological Co., Ltd, Beijing, China). The experiments were carried out at Animal Core Facility of Nanjing Medical University. All procedures were approved by the Experimental Animal Care and Use Committee of Nanjing Medical University and conducted in accordance with the Guide for the Care and Use of Laboratory Animals (NIH publication no. 85-23, revised 1996). The rats were kept in a temperature-controlled room with a 12-h light–dark cycle with free access to standard chow and tap water.

### MI rat model

The MI rat model in the present study was induced by coronary artery ligation using sterile techniques as reported in a previous study [22]. Briefly, the rats were anesthetized with isoflurane (2.5%). A ventilator connection with the gas anesthesia machine was used during establishing the MI rat model. Rats were randomly subjected to the ligation of the left anterior descending coronary artery and sham operation (Sham). The heart was exposed through a left intercostal thoracotomy, and the left coronary artery was looped with a single nylon suture. Finally, the heart was quickly repositioned into the chest. The rats in the sham group were treated in the same way as the rats with coronary ligation, except that their coronary arteries were not ligated.

### Animal grouping

Rats were subjected to MI or Sham. At the same time, apelin-13 (10 nmol/kg/day, i.p., Phoenix Pharmaceuticals, CA, U.S.A.) [23] or saline was administered for 28 days. After 28-day treatment with apelin-13, transthoracic echocardiography, and hemodynamic monitoring were performed then killed with an overdose of pentobarbital (100 mg.kg^−1^, I.V.). The left ventricle (LV) was sectioned for Masson’s staining and the remaining tissue was used for quantitative reverse-transcription PCR (qRT-PCR) and Western blooting.

### Echocardiography

After 4 weeks of MI and apelin-13 treatment, transthoracic echocardiography was performed under isoflurane anesthesia using an ultrasound (Vevo 2100, VisualSonics, Toronto, Canada) with a 21-MHz probe. The left ventricular end-systolic diameter (LVESD), end-diastolic diameter (LVEDD), LV volumes in systole (LVVs) and diastole (LVVd), and LV mass were measured. The LV ejection fraction (EF) and fractional shortening (FS) were calculated. Measurements over three consecutive cardiac cycles were averaged.

### Hemodynamic monitoring

The rats were anesthetized with isoflurane (2.5%), and a 1.4-F conductance micromanometer-tip catheter (Millar Instruments, TX, U.S.A.) was inserted via the right carotid artery across the aortic valve and into the LV chamber. The LV end-diastolic pressure (LVEDP), LV systolic pressure (LVSP), and maximum of the first derivative of LV pressure (LV+d*P*/d*t*_max_) were obtained on a PowerLab data acquisition system (AD Instruments, Sydney, Australia).

### Masson’s trichrome staining

The rats were killed with an overdose of pentobarbital (100 mg/kg, I.V.). The hearts were removed after PBS perfusion. Sections of the LV (5 µm) were examined by Masson’s trichrome staining (Biochannel Biotechnology Co., Ltd., Nanjing, China) according to the manufacturer’s protocol to determine the extent of fibrosis. Tissue sections from rat hearts were observed under a light microscope (Zeiss, Oberkochen, Germany). Images were analyzed using the Image-Pro Plus software (Media Cybernetics, Inc., MD, U.S.A.).

### Culture of CFs isolated from adult rats

Adult CFs were obtained from male SD rats using two independent isolation procedures. The ventricular tissue was dissected, washed, minced, and subjected to seven-times repeated digestions at 37°C for 20 min in a solution containing a mixture of 1  mg/ml of collagenase A and 0.5  mg/ml hyaluronidase after an initial digestion step in a proteinase bacterial solution (4  U/ml) for 15 min. After each cycle of digestion, the tissue was mechanically dissociated using a wide-mouth pipette, the supernatant containing dissociated cells was collected, and the cells were resuspended in Iscove’s modified Dulbecco’s medium (IMDM). The cells from all digestions were pooled and resuspended in IMDM supplemented with 20% fetal bovine serum, penicillin (100 units/ml), streptomycin (100 μg/ml), nonessential amino acids (1%), and 2-mercaptoethanol (0.1 mM). The cells were plated and incubated for 2 h to allow for the preferential attachment of fibroblasts. CFs were used for experiments between passages 3 and 5. CFs were incubated with Ang II (10^−6^ M, Sigma, MO, U.S.A.) for 24 h to induce the fibrotic phenotype. CFs were assigned to four groups, including PBS group, Ang II group, Apelin-13 group, Ang II+Apelin-13 (10 μM) group.

### Western blot analysis

The LV or cultured CFs were sonicated in RIPA lysis buffer and homogenized. The debris was removed, and the supernatant was obtained by centrifugation at 12000×***g*** for 10 min at 4°C. Approximately 30–50 μg protein was separated by electrophoresis, transferred to a PVDF membrane, and probed with primary antibodies against collagen I, collagen III, transforming growth factor-β (TGF-β), and α-smooth muscle actin (SMA) (Abcam, MA, U.S.A.); PI3K, Akt, and p-Akt (Cell Signaling Technology, MA, U.S.A.); and glyceraldehyde-3-phosphate dehydrogenase (GAPDH, Abcam) was used as an internal control. Images were analyzed using the Image-Pro Plus software.

### Real-time polymerase chain reaction

RNA was isolated from cultured CFs using TRIzol (Thermo Fisher Scientific, Shanghai, China). Total RNA (0.5 μg) was reverse transcribed to cDNA. Real-time polymerase chain reactions were performed on an ABI Prism 7900 system. TaqMan probes to detect apelin, APJ, collagen I, collagen III, TGF-β, and α-SMA were purchased from Roche. All samples were amplified in triplicates for 45 cycles in a 384-well plate. The relative level of mRNA expression was expressed as 2^−ΔΔ*C*_t_^. The primers are shown in Table 1.

**Table 1:** Primers used for qRT-PCR

Gene	Species	Forward primer	Reverse primer
*Collagen I*	Rat	TCAAGATGGTGGCCGTTAC	CTGCGGATGTTCTCAATCTG
*Collagen III*	Rat	CGAGATTAAAGCAAGAGGAA	GAGGCTTCTTTACATACCAC
*α-SMA*	Rat	GTCCCAGACATCAGGGAGTAA	TCGGATACTTCAGCGTCAGGA
*TGF-β*	Rat	CAGGGAGTAAGGGACACGA	ACAGCAGTTAGGAACCCAGAT
*GAPDH*	Rat	GGCACAGTCAAGGCTGAGAATG	ATGGTGGTGAAGACGCCAGTA

### Measurement of NADPH oxidase activity

The NAD(P)H oxidase activity in the heart was measured by enhanced lucigenin chemiluminescence. Briefly, the LV or CFs were sonicated in RIPA lysis buffer and homogenized. The debris was removed, and the supernatant was obtained by centrifugation at 12000×***g*** for 10 min at 4°C. NAD(P)H (100 μM) was added to the supernatant as a substrate to react with NAD(P)H oxidase and generate superoxide anions. The light emission produced by the reaction of lucigenin (5 μM) with superoxide anions was measured with a microplate reader (BioTek, VT, U.S.A.) once every minute for 10 min. The values represented the NAD(P)H oxidase activity and were expressed as the mean light units (MLUs) per minute per milligram of protein. The total protein in the supernatant was measured by a BCA protein assay kit (BioChannel Biotechnology Co., Ltd, Nanjing, China).

### Measurement of superoxide anions

Superoxide anions level in the heart was determined by lucigenin-derived chemiluminescence. Briefly, the LV or CFs were sonicated in RIPA lysis buffer and homogenized. The debris was removed, and the supernatant was obtained by centrifugation at 12000×***g*** for 10 min at 4°C. The reaction with superoxide anions was started by adding dark-adapted lucigenin (5 μM) to each supernatant to cause photon emission, which was measured with a microplate reader (BioTek, VT, U.S.A.) once every minute for 10 min. The values representing the superoxide anions level were expressed as the MLU per minute per milligram of protein. The total protein in the supernatant was measured by a BCA protein assay kit (BioChannel Biotechnology Co., Ltd, Nanjing, China).

### PI3K overexpression

For overexpression experiments, adenovirus carrying PI3K (Ad-PI3K) coding sequence (GeneChem, Shanghai, China) was diluted in PBS and added into the medium. The adenovirus carrying green fluorescent protein (GFP, Ad-GFP) was used as a control. Generally, CFs were infected with adenovirus at 50 multiplicity of infection (MOI) for 24 h.

### Statistical analyses

Data were presented as mean ± standard error of the mean. Using GraphPad Prism 5.0 (GraphPad software Inc., CA, U.S.A.), statistical significance among multiple groups was evaluated by one-way analysis of variance with the Bonferroni post-hoc test. A two-tailed *P*-value <0.05 was considered statistically significant.

## Results

### Expression levels of apelin and APJ in the heart of MI rats

**Figure 1:**
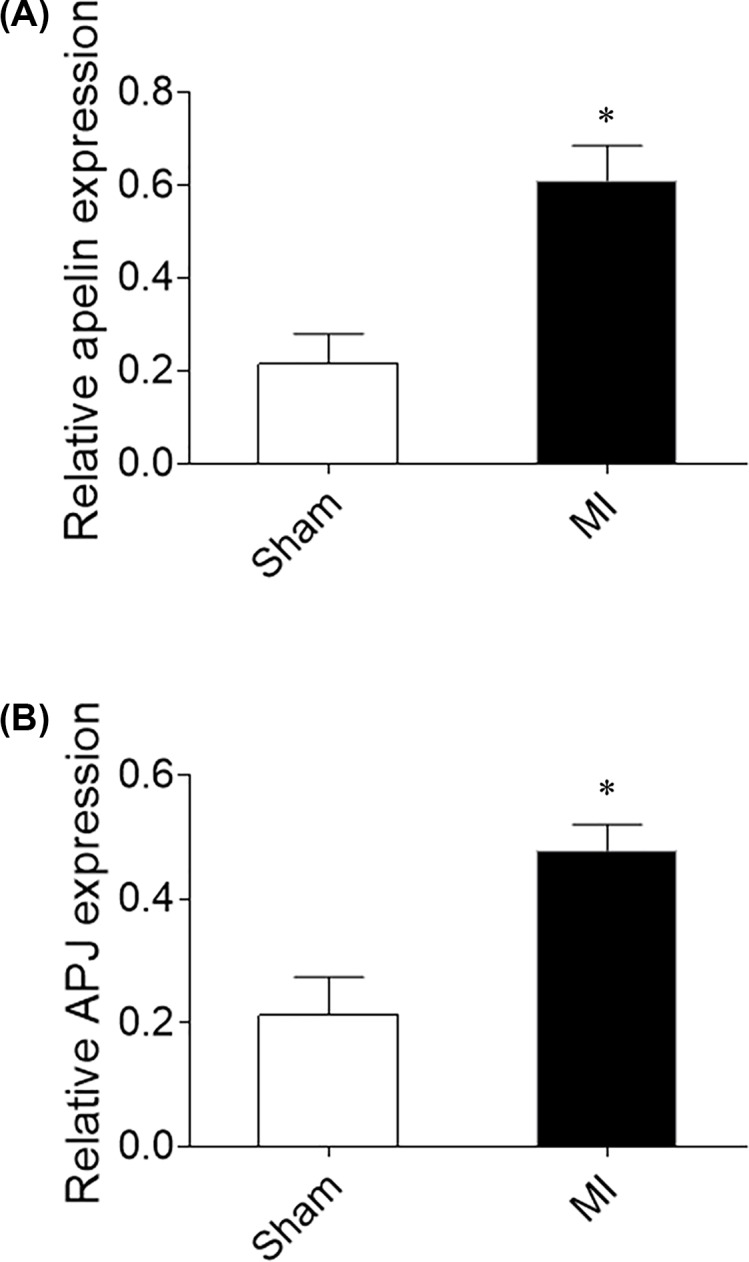
Apelin and APJ expression in the heart of MI rats (**A**) The expression of apelin was increased in the heart of MI rats. (**B**) The expression of APJ was increased in the heart of MI rats. The results are expressed as mean ± standard error (*n*=8). *P<0.05 versus the Sham group.

The expression of apelin in the heart of MI rats was higher than sham rats. In addition, the level of APJ was increased in the heart of MI rats (Figure 1).

### Effects of apelin-13 on cardiac function in rats with MI

**Figure 2:**
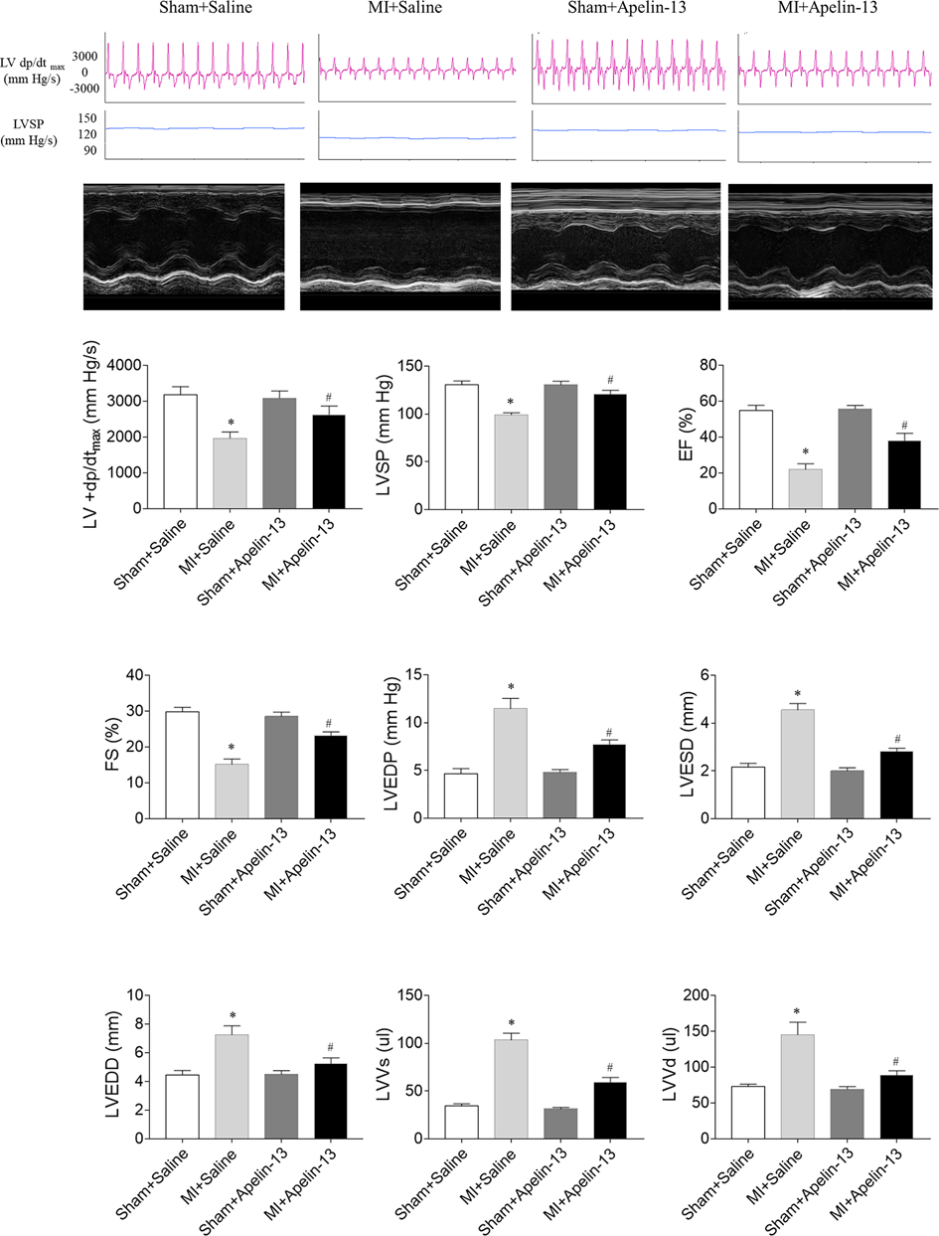
Effects of apelin-13 on cardiac function in rats with MI Apelin-13 reversed MI-induced the decreases in the maximum of the first derivative of left ventricular pressure (LV +d*P*/d*t*_max_), LVSP, EF and FS, and the increases in the LVEDP, LVESD, LVEDD, LVVs, LVVd. The results are expressed as mean ± standard error (*n*=8). *P<0.05 versus the Sham + Saline group; ^#^*P*<0.05 versus the MI + Saline group.

In rats with MI, LV+d*P*/d*t*_max_, LVSP, EF, and FS were reduced, and apelin-13 treatment enhanced the decreases of LV+ d*P*/d*t*_max_, LVSP, EF, and FS in rats with MI. LVEDP, LVESD, LVEDD, LVVs, and LVVd increased in rats with MI, which were reversed by apelin-13 treatment (Figure 2).

### Effects of apelin-13 on cardiac fibrosis in rats with MI

**Figure 3:**
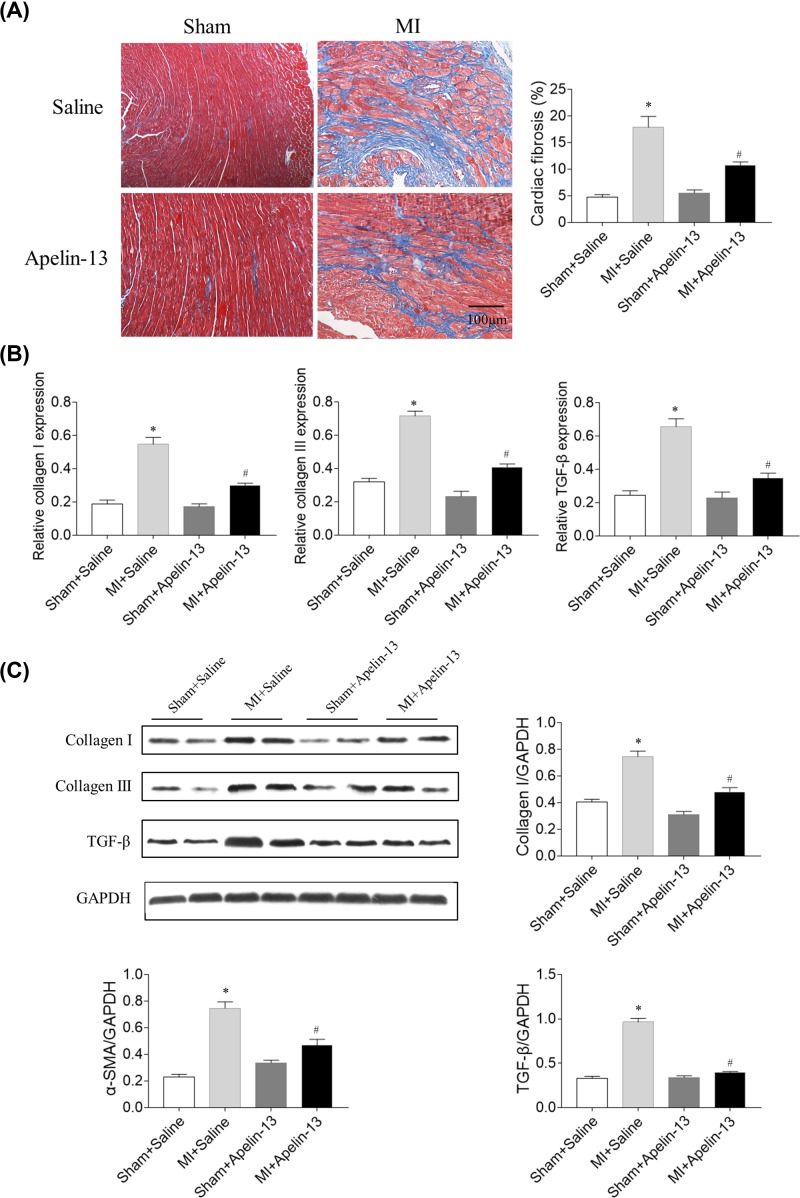
Effects of apelin-13 on cardiac fibrosis in rats with MI (**A**) Apelin-13 attenuated cardiac fibrosis (blue) in MI rats. (**B**) Apelin-13 reduced the mRNA levels of collagen I, collagen III, and TGF-β in the heart of MI rats. (**C**) Apelin-13 reduced the protein levels of collagen I, collagen III, and TGF-β in the heart of MI rats. The results are expressed as mean ± standard error (*n*=8). *P<0.05 versus the Sham + Saline group; ^#^*P*<0.05 versus the MI + Saline group.

The Masson’s staining results showed that cardiac fibrosis increased in rats with MI, which was prevented by apelin-13 (Figure 3A). The mRNA levels of collagen I, collagen III, and TGF-β increased in the heart of rats with MI, which was inhibited by apelin-13 treatment (Figure 3B). The protein levels of collagen I, collagen III, and TGF-β increased in the heart of MI rats, and apelin-13 treatment attenuated the increases in the protein levels of collagen I, collagen III, and TGF-β in the heart of MI rats (Figure 3C).

### Effects of apelin-13 on fibrosis in CFs

**Figure 4:**
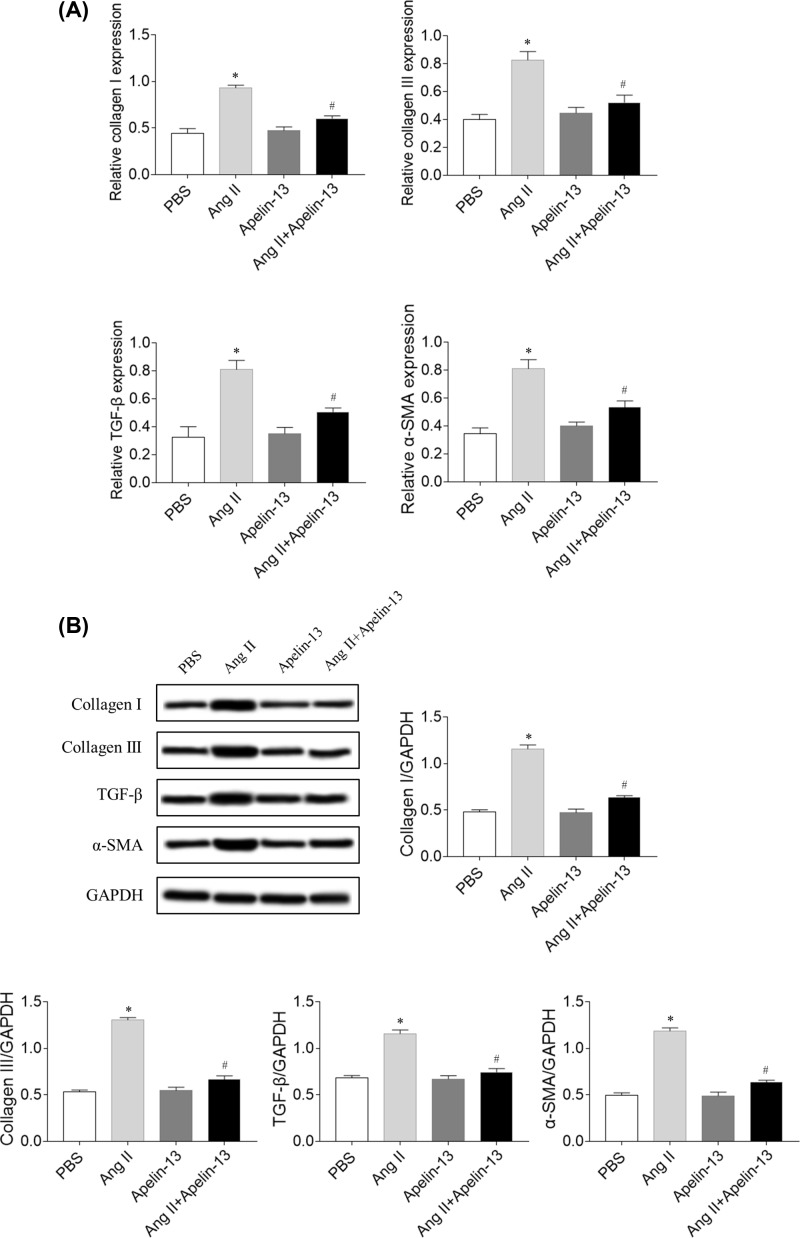
Effects of apelin-13 on fibrosis induced by Ang II in CFs (**A**) Apelin-13 reduced the mRNA levels of collagen I, collagen III, TGF-β, and SMA in the CFs induced by Ang II. (**B**) Apelin-13 reduced the protein levels of collagen I, collagen III, TGF-β, and SMA in the CFs induced by Ang II. The results are expressed as mean ± standard error. *P<0.05 versus the PBS group; ^#^*P*<0.05 versus the Ang II group.

The mRNA expression levels of collagen I, collagen III, TGF-β, and α-SMA were higher in the Ang II group compared with the phosphate-buffered saline (PBS) group in CFs. Treatment with apelin-13 inhibited the increases in the mRNA levels of collagen I, collagen III, TGF-β, and α-SMA induced by Ang II administration in CFs (Figure 4A). The protein levels of collagen I, collagen III, TGF-β, and α-SMA were higher in the Ang II group, which was inhibited by apelin-13 treatment in CFs (Figure 4B).

### Levels of PI3K/Akt

**Figure 5:**
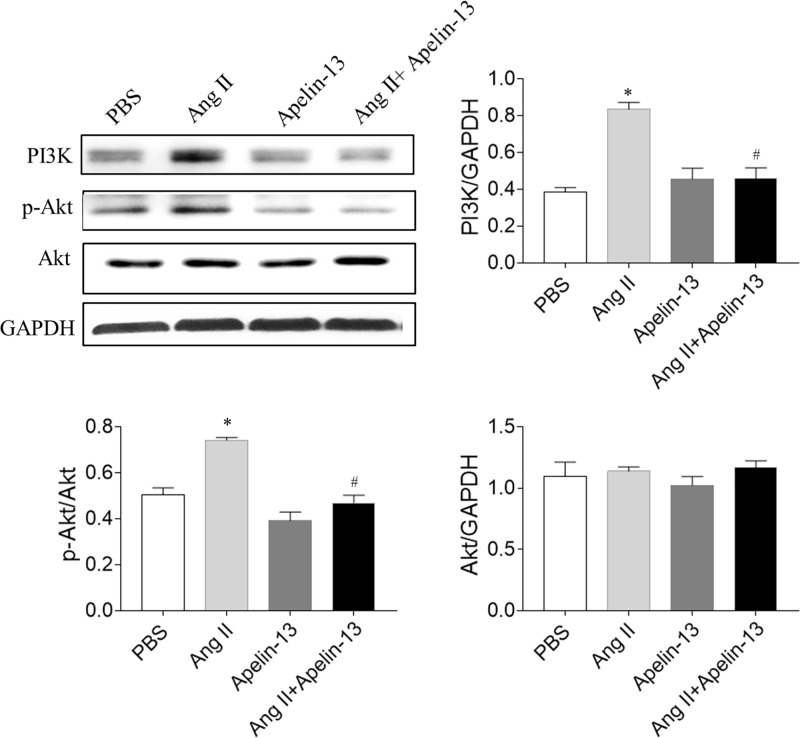
Levels of PI3K/Akt signaling molecules The levels of PI3K and p-Akt were increased in Ang II-treated CFs, and these increases were blocked by apelin-13. The results are expressed as mean ± standard error. *P<0.05 versus the PBS group; ^#^*P*<0.05 versus the Ang II group.

The levels of PI3K and p-Akt increased in Ang II-treated CFs, and these increases were blocked by apelin-13CFs. However, no significant difference in the Akt level was found in the four groups (Figure 5).

### Effects of PI3K overexpression

**Figure 6:**
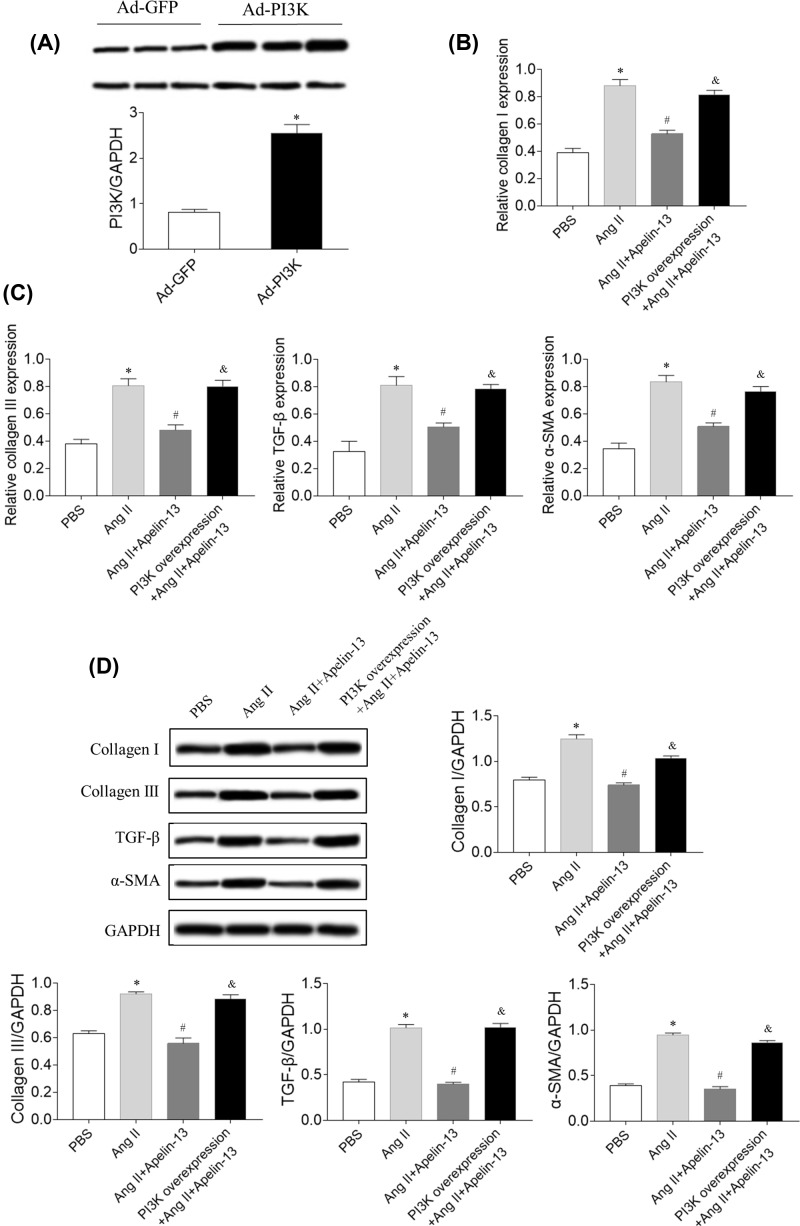
Effects of PI3K overexpression (**A**) The level of PI3K was increased in CFs treated with Ad-PI3K. (**B,C**) The increases in the mRNA levels of collagen I, collagen III, TGF-β, and α-SMA induced by Ang II were reversed after PI3K overexpression in CFs. (**D**) The increases in the protein levels of collagen I, collagen III, TGF-β, and SMA induced by Ang II were reversed after PI3K overexpression in CFs. The results are expressed as mean ± standard error. *P<0.05 versus the PBS group; ^#^*P*<0.05 versus the Ang II group; ^&^*P*<0.05 versus the Ang II + apelin-13 group.

PI3K expression level in Ad-PI3K-treated CFs was 3.14-times of that in control CFs (Figure 6A). PI3K overexpression reversed the effects of apelin-13 on Ang II-induced increases in the mRNA levels of collagen I, collagen III, TGF-β, and α-SMA in CFs (Figure 6B,C). Furthermore, PI3K overexpression reversed the effects of apelin-13 on Ang II-induced increases in the protein levels of collagen I, collagen III, TGF-β, and α-SMA in CFs (Figure 6D).

### Effects of apelin-13 on ROS in CFs

**Figure 7:**
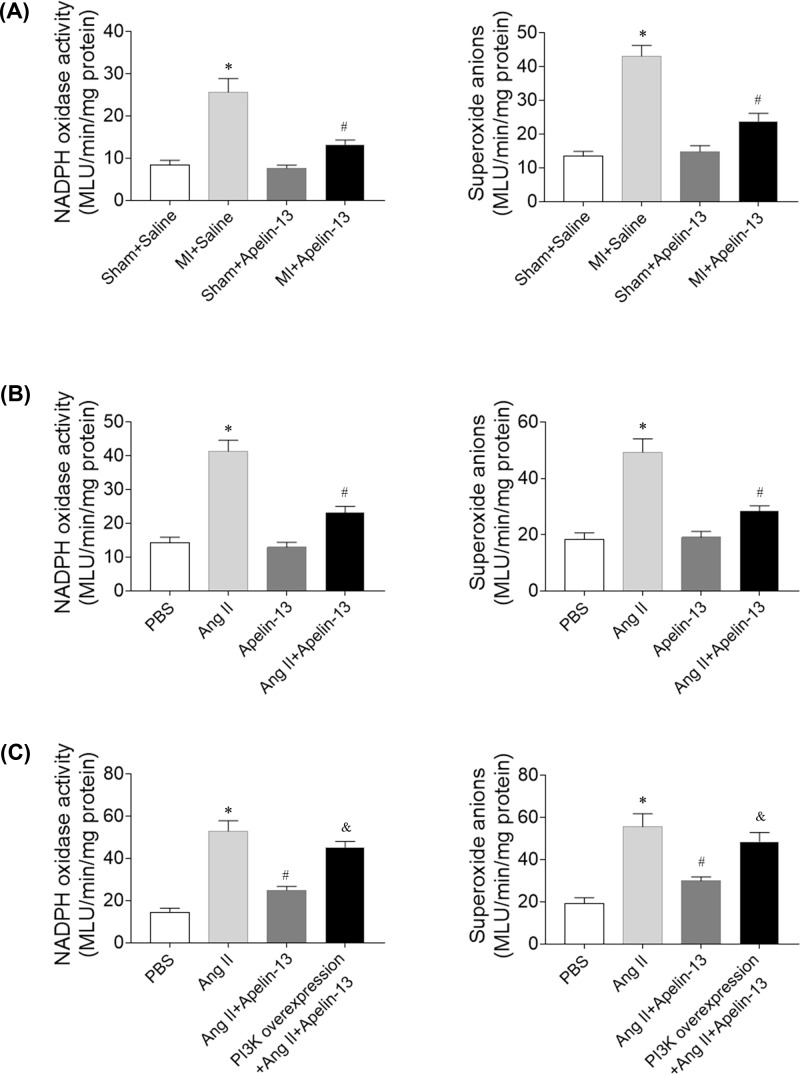
Effects of apelin-13 on reactive oxygen species levels (**A**) The increases in the levels of NADPH oxidase activity and superoxide anions induced by Ang II were reduced after apelin-13 treatment in the heart of MI rats. (**B**) The increases in the levels of NADPH oxidase activity and superoxide anions induced by Ang II were reduced after apelin-13 treatment in the CFs. (**C**) The PI3K overexpression reversed the effects of apelin-13 on Ang II-induced increases in the levels of NADPH oxidase activity and superoxide anions in CFs. The results are expressed as mean ± standard error. *P<0.05 versus the Sham + Saline group (A) or PBS group (B,C); ^#^*P*<0.05 versus the MI + Saline group (A) or Ang II group (B,C); ^&^*P*<0.05 versus the Ang II + apelin-13 group.

NADPH oxidase activity and superoxide anions levels were higher in the heart of MI rats. Apelin-13 reduced the increases in the levels of NADPH oxidase activity and superoxide anions in the heart of MI rats (Figure 7A). The levels of NADPH oxidase activity and superoxide anions were higher in CFs treatment with Ang II, and these increases were blocked by apelin-13 administration (Figure 7B). The PI3K overexpression reversed the effects of apelin-13 on Ang II-induced increases in the levels of NADPH oxidase activity and superoxide anions in CFs (Figure 7C).

## Discussion

CHF is preceded by ventricular remodeling, and cardiac fibrosis is a major driver of disease progression in CHF [4]. Excessive fibrosis causes large infarct scars, resulting in cardiac dilatation and cardiac dysfunction [5,6]. Apelin is a detrimental mechanism that promotes liver fibrosis mainly via up-regulating the expression of collagen I and platelet-derived growth factor receptor β. On the contrary, apelin is beneficial for renal fibrosis and pulmonary fibrosis [24]. The present study showed that apelin-13 improved cardiac dysfunction, impaired cardiac hemodynamics, and attenuated fibrosis of CFs in HF via inhibiting the PI3K/Akt signaling pathway.

In rats with left coronary artery ligation, the LV function reduced as indicated by the decreases in EF, infarct thickness, ± LV d*P*/d*t*, LV developed pressure, and end-diastolic pressure [25]. Apelin in the hypothalamic paraventricular nucleus can improve the cardiac function of rats with thoracic surgical trauma [26]. (3*R*)-5,6,7-Trihydroxy-3-isopropyl-3-methylisochroman-1-one protected cardiomyocytes against isoproterenol-induced MI, potentially via the apelin/apelin receptor signaling pathway [27]. The apelin/APJ system is vital in the regulation of myocardial contractility and blood pressure [27]. In the present study, the results showed that LV +d*P*/d*t*_max_, LVSP, EF, and FS reduced in rats with MI, and apelin-13 treatment enhanced the decreases in LV +d*P*/d*t*_max_, LVSP, EF, and FS. LVEDP, LVESD, LVEDD, LVVs, and LVVd increased in rats with MI, which were reversed by apelin-13 treatment. These results indicated that apelin-13 improved cardiac dysfunction and impaired cardiac hemodynamics in rats with HF, which is supported by previous study that apelin-13 treatment improved left ventricular function of MI rats [28].

Cardiac remodeling is an important mechanism for the occurrence and development of CHF [29]. CFs played a key role in postinfarction remodeling, which can ultimately lead to pathological fibrosis and HF [8]. Apelin ameliorated the expression of Ang II-induced TGF-β in primary cardiomyocytes, accompanied by reduced hypertrophy [30]. The present study found that the expression levels of collagen I, TGF-β, and α-SMA increased in the hearts of rats with MI, which were inhibited by apelin-13 treatment. The mRNA levels of collagen I, collagen III, TGF-β, and α-SMA were higher in the Ang II group compared with the PBS group in CFs. Treatment with apelin-13 inhibited the increases in the mRNA levels of collagen I, collagen III, TGF-β, and α-SMA induced by Ang II administration in CFs. These results demonstrated that the increase in cardiac fibrosis in rats with HF was attenuated by apelin-13 treatment.

MI was associated with decreased activities of PI3K and signal transducer and activator of transcription 3 (STAT3) in aging rats compared with young rats [31]. The Akt signaling pathway was enhanced in CFs after Ang II treatment [32]. Apelin-13 blocked cisplatin-induced H9C2 cell apoptosis via the regulation of mitogen-activated protein kinases (MAPKs) and PI3K/Akt signaling pathway [33]. The present study showed that the levels of PI3K and p-Akt increased in Ang II-treated CF, and these increases were blocked by apelin-13. PI3K overexpression reversed the effects of apelin-13 on Ang II-induced increases in the mRNA levels of collagen I, collagen III, TGF-β, and α-SMA in CFs. The results indicated that apelin-13 alleviated fibrosis induced by Ang II via inhibiting the PI3K/Akt signaling pathway. However, previous study revealed that apelin-13 promoted the phosphorylation of PI3K and Akt to induce cardiomyocyte hypertrophy of H9C2 [20]. These findings indicated that apelin-13 showed protective effects in CFs on attenuating fibrosis via inhibiting PI3K/Akt pathway; while apelin-13 had adverse effects in cardiomyocytes on promoting hypertrophy via enhancing PI3K/Akt signaling.

Oxidative stress, defined as an excess production of reactive oxygen species (ROS), has been shown to play important roles in the pathophysiology of HF and cardiac remodeling [34]. Apelin/APJ played important roles in oxidative stress-related inflammatory diseases [35]. Whether apelin can attenuate HF via inhibiting ROS is not well known. In the present study, NADPH oxidase activity and superoxide anions levels were higher in the heart of MI rats and in CFs treatment with Ang II, and these increases were blocked by apelin-13 administration. The PI3K overexpression reversed the effects of apelin-13 on Ang II-induced increases in NADPH oxidase activity and superoxide anions in CFs. The results demonstrated that apelin-13 attenuated oxidative stress via inhibiting the PI3K/Akt signaling pathway.

In conclusion, apelin-13 improved cardiac dysfunction, attenuated impaired cardiac hemodynamics, and alleviated fibrosis in rats with HF, and apelin-13 attenuated fibrosis of CFs induced by Ang II via inhibiting the PI3K/Akt signaling pathway to attenuate oxidative stress.

## Abbreviations

Akt: protein kinase B
Ad-PI3K: adenovirus carrying PI3K
Ang: angiotensin
CF: cardiac fibroblast
CHF: chronic heart failure
EF: ejection fraction
FS: fractional shortening
GFP: green fluorescent protein
HF: heart failure
IMDM: Iscove’s modified Dulbecco’s medium
I.P.: intraperitoneally
I.V.: intravenous
LV: left ventricular
LVEDD: left ventricular end-diastolic diameter
LVEDP: LV end-diastolic pressure
LVESD: left ventricular end-systolic diameter
LVSP: LV systolic pressure
LVVd: left ventricular volume in diastole
LVVs: left ventricular volume in systole
MI: myocardial infarction
MLU: mean light unit
PI3K: phosphatidylinositol 3-kinase
RIPA: radio immunoprecipitation assay
SD: Sprague–Dawley
SMA: smooth muscle actin
STAT3: signal transducer and activator of transcription 3
TGF: transforming growth factor

## References

[1] Go A.S., Mozaffarian D., Roger V.L. et al. (2014) Heart disease and stroke statistics–2014 update: a report from the American Heart Association. Circulation 129, e28–e292 24352519 10.1161/01.cir.0000441139.02102.80 PMC5408159

[2] Raso A., Dirkx E., Philippen L.E. et al. (2019) Therapeutic delivery of miR-148a suppresses ventricular dilation in heart failure. Mol. Ther. 27, 584–599 10.1016/j.ymthe.2018.11.011 30559069 PMC6403487

[3] Gan X.T., Ettinger G., Huang C.X. et al. (2014) Probiotic administration attenuates myocardial hypertrophy and heart failure after myocardial infarction in the rat. Circ. Heart Fail. 7, 491–499 10.1161/CIRCHEARTFAILURE.113.000978 24625365

[4] Tarone G., Balligand J.L., Bauersachs J. et al. (2014) Targeting myocardial remodelling to develop novel therapies for heart failure: a position paper from the Working Group on Myocardial Function of the European Society of Cardiology. Eur. J. Heart Fail. 16, 494–508 10.1002/ejhf.62 24639064

[5] Kong P., Christia P. and Frangogiannis N.G. (2014) The pathogenesis of cardiac fibrosis. Cell. Mol. Life Sci. 71, 549–574 10.1007/s00018-013-1349-6 23649149 PMC3769482

[6] Heineke J. and Molkentin J.D. (2006) Regulation of cardiac hypertrophy by intracellular signalling pathways. Nat. Rev. Mol. Cell Biol. 7, 589–600 10.1038/nrm1983 16936699

[7] Tallquist M.D. and Molkentin J.D. (2017) Redefining the identity of cardiac fibroblasts. Nat. Rev. Cardiol. 14, 484–491 10.1038/nrcardio.2017.57 28436487 PMC6329009

[8] Philip J.L., Xu X., Han M. et al. (2019) Regulation of cardiac fibroblast-mediated maladaptive ventricular remodeling by beta-arrestins. PLoS ONE 14, e0219011 10.1371/journal.pone.0219011 31269046 PMC6609028

[9] Yang P., Kuc R.E., Brame A.L. et al. (2017) [Pyr(1)]Apelin-13(1-12) is a biologically active ACE2 metabolite of the endogenous cardiovascular peptide [Pyr(1)]Apelin-13. Front. Neurosci. 11, 92 10.3389/fnins.2017.00092 28293165 PMC5329011

[10] Wysocka M.B., Pietraszek-Gremplewicz K. and Nowak D. (2018) The role of apelin in cardiovascular diseases, obesity and cancer. Front. Physiol. 9, 557 10.3389/fphys.2018.00557 29875677 PMC5974534

[11] Harford-Wright E. and Gavard J. (2018) Apelin, the devil inside brain tumors. J. Exp. Neurosci. 12, 1179069518759680, 10.1177/1179069518759680 29535551 PMC5843094

[12] Fan X.F., Xue F., Zhang Y.Q. et al. (2015) The Apelin-APJ axis is an endogenous counterinjury mechanism in experimental acute lung injury. Chest 147, 969–978 10.1378/chest.14-1426 25375801

[13] Tatemoto K., Hosoya M., Habata Y. et al. (1998) Isolation and characterization of a novel endogenous peptide ligand for the human APJ receptor. Biochem. Biophys. Res. Commun. 251, 471–476 10.1006/bbrc.1998.9489 9792798

[14] Leung O.M., Li J., Li X. et al. (2018) Regulatory T cells promote apelin-mediated sprouting angiogenesis in type 2 diabetes. Cell Rep. 24, 1610–1626 10.1016/j.celrep.2018.07.019 30089270

[15] O’Carroll A.M., Lolait S.J., Harris L.E. et al. (2013) The apelin receptor APJ: journey from an orphan to a multifaceted regulator of homeostasis. J. Endocrinol. 219, R13–R35 10.1530/JOE-13-0227 23943882

[16] Nishida M., Okumura Y., Oka T. et al. (2012) The role of apelin on the alleviative effect of Angiotensin receptor blocker in unilateral ureteral obstruction-induced renal fibrosis. Nephron Extra 2, 39–47 10.1159/000337091 22619666 PMC3350347

[17] Melgar-Lesmes P., Casals G., Pauta M. et al. (2010) Apelin mediates the induction of profibrogenic genes in human hepatic stellate cells. Endocrinology 151, 5306–5314 10.1210/en.2010-0754 20843995

[18] Yang W., Wu Z., Yang K. et al. (2019) BMI1 promotes cardiac fibrosis in ischemia-induced heart failure via the PTEN-PI3K/Akt-mTOR signaling pathway. Am. J. Physiol. Heart Circ. Physiol. 316, H61–H69 10.1152/ajpheart.00487.2018 30359076

[19] Liu F., Wen Y., Kang J. et al. (2018) Regulation of TLR4 expression mediates the attenuating effect of erythropoietin on inflammation and myocardial fibrosis in rat heart. Int. J. Mol. Med. 42, 1436–1444 29845292 10.3892/ijmm.2018.3707 PMC6089778

[20] Xie F., Liu W., Feng F. et al. (2015) Apelin-13 promotes cardiomyocyte hypertrophy via PI3K-Akt-ERK1/2-p70S6K and PI3K-induced autophagy. Acta Biochim. Biophys. Sin. (Shanghai) 47, 969–980 10.1093/abbs/gmv111 26607438

[21] Ohtsu H., Frank G.D., Utsunomiya H. et al. (2005) Redox-dependent protein kinase regulation by angiotensin II: mechanistic insights and its pathophysiology. Antioxid. Redox Signal. 7, 1315–1326 16115037 10.1089/ars.2005.7.1315

[22] Gan X.B., Duan Y.C., Xiong X.Q. et al. (2011) Inhibition of cardiac sympathetic afferent reflex and sympathetic activity by baroreceptor and vagal afferent inputs in chronic heart failure. PLoS ONE 6, e25784 10.1371/journal.pone.0025784 21991351 PMC3185007

[23] Azizi Y., Imani A., Fanaei H. et al. (2017) Post-infarct treatment with [Pyr1]apelin-13 exerts anti-remodelling and anti-apoptotic effects in rats’ hearts. Kardiol. Pol. 75, 605–613 10.5603/KP.a2017.0022 28181211

[24] Huang S., Chen L., Lu L. et al. (2016) The apelin-APJ axis: a novel potential therapeutic target for organ fibrosis. Clin. Chim. Acta 456, 81–88 10.1016/j.cca.2016.02.025 26944568

[25] Zhou Y., Richards A.M. and Wang P. (2019) MicroRNA-221 is cardioprotective and anti-fibrotic in a rat model of myocardial infarction. Mol. Ther. Nucleic Acids 17, 185–197 10.1016/j.omtn.2019.05.018 31261033 PMC6606926

[26] Zhang H.H., Wang Y.J., Zheng C. et al. (2018) Apelin in the hypothalamic paraventricular nucleus improves cardiac function in surgical trauma rats. Sheng Li Xue Bao [Acta Physiol. Sin.] 70, 99–105 29691573

[27] Yeganeh-Hajahmadi M., Najafipour H., Farzaneh F. et al. (2018) Effect of apelin on cardiac contractility in acute reno-vascular hypertension: The role of apelin receptor and kappa opioid receptor heterodimerization. Iran J. Basic Med. Sci. 21, 1305–1315 30627376 10.22038/IJBMS.2018.31361.7555 PMC6312676

[28] Zhang X., Hu W., Feng F. et al. (2016) Apelin-13 protects against myocardial infarction-induced myocardial fibrosis. Mol. Med. Rep. 13, 5262–5268 10.3892/mmr.2016.5163 27109054

[29] Liu M., Ai J., Feng J. et al. (2019) Effect of paeoniflorin on cardiac remodeling in chronic heart failure rats through the transforming growth factor beta1/Smad signaling pathway. Cardiovasc. Diagn. Ther. 9, 272–280 10.21037/cdt.2019.06.01 31275817 PMC6603499

[30] Sato T., Kadowaki A., Suzuki T. et al. (2019) Loss of Apelin augments angiotensin II-induced cardiac dysfunction and pathological remodeling. Int. J. Mol. Sci. 20, 239 10.3390/ijms20020239 PMC6358887 30634441

[31] Lin C.C., Chen S.Y., Lien H.Y. et al. (2019) Targeting the PI3K/STAT3 axis modulates age-related differences in macrophage phenotype in rats with myocardial infarction. J. Cell. Mol. Med. 23, 6378–6392 10.1111/jcmm.14526 31313516 PMC6714172

[32] Wang L., Liu C., Chen X. et al. (2019) Alamandine attenuates longterm hypertensioninduced cardiac fibrosis independent of blood pressure. Mol. Med. Rep. 19, 4553–4560 31059021 10.3892/mmr.2019.10167 PMC6522836

[33] Zhang P., Yi L.H., Meng G.Y. et al. (2017) Apelin-13 attenuates cisplatin-induced cardiotoxicity through inhibition of ROS-mediated DNA damage and regulation of MAPKs and AKT pathways. Free Radic. Res. 51, 449–459 10.1080/10715762.2017.1313414 28554248

[34] Tsutsui H., Kinugawa S. and Matsushima S. (2011) Oxidative stress and heart failure. Am. J. Physiol. Heart Circ. Physiol. 301, H2181–H2190 10.1152/ajpheart.00554.2011 21949114

[35] Zhou Q., Cao J. and Chen L. (2016) Apelin/APJ system: a novel therapeutic target for oxidative stress-related inflammatory diseases (Review). Int. J. Mol. Med. 37, 1159–1169 10.3892/ijmm.2016.2544 27035220

